# Flavanol-Rich Cocoa Powder Interacts with *Lactobacillus* *rhamnossus* *LGG* to Alter the Antibody Response to Infection with the Parasitic Nematode *Ascaris suum*

**DOI:** 10.3390/nu9101113

**Published:** 2017-10-12

**Authors:** Saebyeol Jang, Sukla Lakshman, Ethiopia Beshah, Yue Xie, Aleksey Molokin, Bryan T. Vinyard, Joseph F. Urban, Cindy D. Davis, Gloria I. Solano-Aguilar

**Affiliations:** 1 USDA, ARS, Beltsville Human Nutrition Research Center, Diet, Genomics, and Immunology Laboratory, Beltsville, MD 20705, USA; newbyeol@gmail.com (S.J.); Sukla.Lakshman@ars.usda.gov (S.L.); Ethiopia.Beshah@ars.usda.gov (E.B.); Aleksey.Molokin@ars.usda.gov (A.M.); Joe.Urban@ars.usda.gov (J.F.U.J.); 2 Department of Parasitology, College of Veterinary Medicine, Sichuan Agricultural University, Chengdu 611130, China; xyue1985@gmail.com; 3 USDA, ARS, Biometrical Consulting Services, Beltsville, MD 20705, USA; Bryan.Vinyard@ars.usda.gov; 4 Office of Dietary Supplements, NIH, Bethesda, MD 20892, USA; davisci@mail.nih.gov

**Keywords:** LGG, cocoa flavanols, porcine model, gut microbiota, type-2 immune response, toll-like receptors

## Abstract

Consumption of the probiotic bacteria *Lactobacillus* *rhamnosus* *LGG* and flavanol-rich cocoa have purported immune modulating effects. This study compared the host response to infection with *Ascaris suum* in three-month-old pigs fed a standard growth diet supplemented with a vehicle control: LGG, cocoa powder (CP) or LGG + CP. Pigs were inoculated with infective *A*. *suum* eggs during Week 5 of dietary treatment and euthanized 17 days later. *Lactobacillus* abundance was increased in pigs fed LGG or LGG + CP. Specific anti-*A*. *suum* IgG2 antibodies were decreased (*p* < 0.05) in LGG + CP-fed pigs compared to pigs fed CP alone. Pigs fed LGG had significantly reduced expression (*p* < 0.05) of Eosinophil peroxidase (*EPX)*, Interleukin 13 (*IL-13)*, Eotaxin 3 (*CCL26)*, Toll-like receptor 2 (*TLR2*), TLR4, and TLR9 and Interleukin-1Beta (*IL1B*) in the tracheal-bronchial lymph node (TBLN) independent of CP treatment. These results suggested that feeding LGG significantly reduced the localized prototypical Th2-related markers of infection with *A*. *suum* in the TBLN. Although feeding CP does not appear to affect the *A*. *suum-*induced Th2-associated cytokine response, feeding LGG + CP reduced anti-*A*. *suum* antibodies and delayed intestinal expulsion of parasitic larvae from the intestine.

## 1. Introduction

Cocoa products are popular foods rich in flavanols such as catechin and epicathechin monomers, and procyanidin oligomers [1]. Health effects associated with cocoa flavanol consumption depend on their bioavailability and their chemical structure [2]. Monomeric flavanols absorbed in the small intestine can be rapidly detected in plasma and body tissues of animals and humans [3,4]. In contrast, flavanol polymers and trimers, and large pro-anthocyanidins appear to be less well absorbed and the beneficial effects depend on intestinal microbial metabolism in the lower bowel [5]. Consumption of cocoa supplemented diets has been shown to influence host inflammation as well as the adaptive immune response, both systemically and in localized tissues. The evidence in humans seems to depend on the inflammatory status of the host [6] with modification of biomarkers associated with improved clinical outcome [7]. Cocoa-supplemented diets also reduced the synthesis of Th2 associated antibodies in two allergy models induced in rats [8]. The varied health benefits associated with consumption of cocoa products in both animal and human models are likely related to differences in dose and composition of cocoa products [2,6,8]. We previously demonstrated that consumption of flavanol-rich cocoa powder (CP) modulated colonic microbial metabolites in tissues, increased the abundance of the endogenous *Lactobacillus* and *Bifidobacterium* species in the intestine, and reduced pro-inflammatory cytokine and Toll-like receptor (TLR) gene expression in intestinal tissues and mesenteric lymph nodes (MLN) of healthy pigs, suggesting that consumption of CP can support intestinal health [4]. 

Infectious disease models have been routinely used to test the health benefits of diet. We studied the infection of pigs with the common intestinal parasite *Ascaris suum* (*A*. *suum*) because it is a zoonotic organism and related to *A*. *lumbricoides*, the most prevalent species of worm parasite in humans worldwide [9]. Pigs inoculated with *A*. *suum* express a classical Th2-associated cytokine response characterized by eosinophilia and increased IL-4, IL-5 and IL-13 gene expression in the liver, lungs and intestines transiently exposed to migrating larvae prior to natural expulsion from the intestine around Days 17–21 after inoculation [10]. There is a characteristic immediate-type hypersensitivity reaction in the lungs and draining lymph nodes of pigs, similar to human asthmatic airway hyper-sensitivity [11], that has been studied for therapeutic treatment alternatives [12]. In our model, the demonstrated prebiotic effect of CP on endogenous pig *Lactobacillus* species [4] was also tested on exogenous *Lactobacillus rhamnosus* (*LGG*) that was fed to pigs infected with *A*. *suum*. Probiotic supplementation with *Lactobacillus rhamnosus* HN001 had previously been shown to decrease the severity of lung responses to *Ascaris* allergens in sensitized pigs [13]. The objective was to determine if feeding CP with and without LGG would modulate the response to parasitic *A*. *suum* larvae migrating through the lung and small intestine. This study specifically examined the systemic and local response to *A suum* in young pigs fed a corn-, alfalfa-, and soy-based diet supplemented with either CP, LGG, LGG + CP or a vehicle-equivalent maltodextrin-fiber control for 4.5 weeks before inoculation with infective *A*. *suum* eggs. 

## 2. Materials and Methods

### 2.1. Dietary Supplements

*Lactobacillus rhamnosus LGG* and a high flavanol-rich CP powder (Acticoa) were kindly provided by Christian Hansen (Hoersholm, Denmark) and Barry-Callebaut (Lebbeke, Belgium), respectively. Detection of major phenolic compounds in CP was determined by 4-dimethyl aminocinnamaldehyde (DMAC) colorimetric method [14] using a B-type proanthocyanidin dimer as equivalent of milligram proanthocyanidins (PACs) and by individual flavanol analysis with ultra-high performance liquid chromatography with high resolution mass spectrometry (UHPLC-HRMS) Orbitrap MS system (Thermo Fisher Scientific, San Jose, CA, USA) using catechin/epicatechin, procyanidin B1 and procyanidin C1 as standards for flavanol monomers, dimers and trimers, respectively. Flavanol tetramers and pentamers were measured using catechin (Sigma Aldrich, St. Louis, MO, USA) as a reference standard with relative response factors as previously described [4,15]. Insoluble and soluble fiber AOAC (Association of Official Agricultural Chemistry) analysis was done by Covance Laboratories (Madison, WI, USA). LGG viability was assessed by culture in MRS-Agar plates (Anaerobe Systems, Morgan Hill, CA, USA).

### 2.2. Animals and Diets

All animal experiments and procedures were conducted in accordance with guidelines established and approved by the Beltsville Area Animal Care and Use Committee under Protocol No. 13-028 approved on 8 November 2013. Thirty-two 3-month-old male pigs from >12 litters were obtained from Oakhill Genetics Farms (Ewing, IL, USA). Pigs were acclimated for 3 weeks at the USDA facility after arrival, fed a basal corn-, alfalfa-, and soy-based diet prepared on site (Table S1) twice per day, and water ad libitum. Pigs with an average weight of 14.6 ± 1.6 kg (mean ± SD) were randomized by weight into four groups of eight animals, each in a 2 × 2 design, housed indoors individually on concrete floors equipped with heat lamps and airflow to maintain a comfortable temperature (24 °C), twelve-hour light/dark cycle and access to water at all times. Pigs were fed a grower diet supplemented daily with: (1) a vehicle/placebo (maltodextrin) containing 1.24 g/day of dextrin and 6.42 g/day of cellulose to match total soluble and insoluble fiber contributed by CP and 1 g of maltodextrin; (2) 1 × 10^10^ cfu LGG in maltodextrin; (3) 26 g of flavanol-rich CP; or (4) LGG + CP for 7 weeks. Lyophilized LGG or the equivalent amount of maltodextrin alone was mixed with 5 mL of phosphate buffered saline (PBS) until dissolved and delivered orally using individual disposable syringes. Cocoa powder (dose of 26 g) or dextrin and cellulose (equivalent to the amount of fiber in the CP) was dissolved in 30 mL of water and the suspension was applied to the dry feed that provided 2702 kcal/day (Table S1). After 4.5 weeks on the diets, pigs were orally inoculated with infective *A*. *suum* eggs (1 × 10^4^) and then continued on the diet treatment until the end of Week 7. The acquisition and preparation of infective *A*. *suum* eggs, oral inoculation, management of pigs, and recovery of fourth-stage larvae (L4) from the intestines were as previously described [16]. All pigs were sacrificed by IV injection with Euthasol (50 mg sodium pentobarbital/kg of body weight) (Virbac Animal Health, Inc., Fort Worth, TX, USA) 17 days after inoculation with *A*. *suum* eggs.

### 2.3. Blood Collection and Processing

Blood samples were collected from all 32 pigs at baseline (Week 0) and Week 7. Serum samples were sent to Antech Diagnostics Laboratory (Morrisville, NC, USA) to run a basic serum chemistry panel including alkaline phosphatase (ALP), alanine aminotransferase (ALT), aspartate aminotransferase (AST), total bilirubin, lactate dehydrogenase (LDH), gamma glutamyltransferase (GGT) and total protein. Serum aliquots and supernatant derived from ileal contents collected at necropsy (Week 7) were saved for detecting IgM, IgG, IgG1 and IgG2 isotype antibodies against *A*. *suum* 3rd to 4th stage larvae (L3/L4) extract by ELISA. Briefly, ELISA plates (Immunolon, Nunc) were coated with 100 µL of 5 µg/mL of *A*. *suum* L3/L4 extract [16] in coating buffer (Sodium Carbonate buffer, pH 9.5). After an overnight incubation at 4 °C, plates were washed six times (0.05% Tween 20 in 1X-PBS) and blocked with 0.05% Tween 20, 0.5% BSA in PBS for 30 min. Plates were washed and used for testing 100 µL of serum diluted at 1:10, 1:100, 1:1000 and 1:3000 (0.05% Tween 20 in PBS). Test samples were run in duplicates and incubated for 2 h at room temperature. After six washes, 100 µL of HRP-conjugated porcine anti-IgM, IgG, IgG1 or IgG2 (Serotec, Raleigh, NC, USA) were added at a 1:10,000 dilution and incubated for one hour. After a final wash, 100 µL of TMB-One substrate solution (Promega, Madison, WI, USA) was added. Relative anti-*Ascaris* L3/L4 specific antibody was determined after reading plates with an optical density (OD) at 450 nm after 20 min. The reaction was stopped by addition of 1 M HCl solution. 

### 2.4. Tissue and Intestinal Sample Processing

Fecal samples were collected before the start of the study (Week 0) and at necropsy (Week 7). All other samples including supernatant from the ileal contents, serum, tracheal/bronchial lymph nodes (TBLN), mesenteric lymph nodes (MLN), jejunum (mid-section of the small intestine) and alveolar macrophage (AM) isolated from the bronchial/alveolar lavage fluid were collected at the end of Week 7. All samples were initially frozen in liquid nitrogen and then kept at −80 °C until further analysis.

### 2.5. Lactobacillus Rhamnosus Abundance

Fecal samples were collected before the start of the study (Week 0), at Week 4.5 prior to inoculation with *A*. *suum* eggs and at Week 7 (end of the study). The abundance of *Lactobacillus rhamnosus* was measured by real time PCR using specific probes [17]. DNA from feces was isolated using the QIAamp DNA stool kit according to the manufacturer’s instructions (Qiagen, Valencia, CA, USA). DNA concentration was determined by NanoDrop (Thermo Fisher Scientific, Wilmington, DE, USA). Briefly, 40 ng of fecal DNA per sample was used as a template for real time PCR amplification using primers and probes that differentially amplify variable regions within the 16S ribosomal DNA specific for *L rhamnosus*. The Ct value generated for LGG was compared to Ct values from a standard curve constructed using a series of dilutions of purified target gene fragments to determine the target gene copy numbers. The values were expressed as log_10_ target gene copy number per gram (cpg) of sample. A fixed amount of a synthetically designed plasmid pUC57-Kan containing a 219 bp snake venom fragment was mixed into samples and used as a positive control for efficiency of DNA extraction. All molecular assays were performed in duplicate using the 7500-ABI PRISM (Perkin Elmer, Foster City, CA, USA). Mean copy numbers of bacterial species in feces were calculated and compared among treatment groups. 

### 2.6. Isolation of Alveolar Macrophage

Alveolar macrophages (AM) were isolated from pigs at necropsy by bronchial/alveolar lavage [18]. Briefly, both lobes of a lung were gravity filled with 500 mL of PBS, followed by massaging for 30 s, and draining of the cell suspension into 50 mL polypropylene tubes. The cells were centrifuged at 2500 rpm for 5 min, washed in PBS and re-suspended in macrophage culture medium (RPMI 1640, 2% porcine serum, 1% non-essential amino acids, 1% sodium pyruvate, 10 mM HEPES, 100 U/mL penicillin, 100 μg/mL streptomycin, 1.5 g/L sodium bicarbonate) and seeded at a density of 5 × 10^6^ cells per well of six-well tissue culture plates and incubated for 24 h. The cell culture supernatants were replaced with fresh macrophage culture medium and treated with either vehicle (DMEM media) or 10 ng/mL LPS for 24 h. The cells were lysed with 1 mL of TRIzol reagent (Invitrogen, Life Technologies, Carlsbad, CA, USA), and frozen for subsequent RNA isolation and analysis of gene expression.

### 2.7. RNA Extraction, cDNA Synthesis, and Real-Time PCR Analysis (RT-PCR)

Frozen (−80 °C) 1 mm^3^ tissue sections of TBLN, MLN, and jejunum were rapidly homogenized in Trizol (Invitrogen, Grand Island, NY, USA). The RNA extraction and cDNA synthesis were performed as previously described [19]. The sequence of probes and primers and running conditions of RT-PCR were obtained from the Porcine Translational Research Database [20]. The RT-PCR was performed using 15 ng/well of cDNA in 15 μL on an ABI 7900 sequence detector system (Applied Biosystems, Foster City, CA, USA). Data for gene expression were normalized to the housekeeping gene *RPL32*, expressed as ΔCt, and a fold change was compared to the control group of pigs which was designated as 1-fold change. The fold change was calculated from the mean difference of the control group. 

### 2.8. Statistical Analysis

The effects of *LGG*, CP, and their interaction were determined by fitting generalized linear mixed-effects models (GLMMs), using SAS/STAT PROC GLIMMIX. Bacterial abundance and parasite counts were modeled using a negative binomial distribution with log link function. The anti-*Ascaris* antibody response was modeled using a normal distribution and identity link function. For gene expression data, ∆C_T_ and ∆∆C_T_ values were calculated by including another factor, with target gene and baseline gene levels, in the normally-distributed Analysis of Variance ANOVA models and specifying contrasts [21]. Correlation between target and baseline genes and stimulant or no stimulant measured on the same pig was modeled selecting among: heterogeneous or homogeneous and compound symmetric or independence covariance structures using the Akaike Information Criteria Corrected (AICC) [22]. A stimulant factor was added to the ANOVA model for AM, with levels LPS or Media only. Fold change of each Diet × Treatment and each Stimulant × Diet × Treatment, relative to baseline (i.e., control and no LGG or stimulant), was calculated as 2^−∆∆C^_T_. Significance of each pair wise comparison was determined using the Šidák comparison wise error rate (CER) to maintain a maximum, α = 0.05, experiment-wise error rate (MEER) for all pair-wise comparisons among experimental condition, Diet × Treatment or Stimulant × Diet × Treatment, means or fold changes. Significance of fold change differences for each pair, i and k, of experimental conditions were determined using contrasts to test H_0_: ∆∆C_Ti_ − ∆∆C_Tk_ = 0. The Pearson correlation coefficient was calculated between *A*. *suum* larvae counts in intestinal lumen and the IgG, IgM, IgG1 and IgG2 OD values. Analysis were carried out using SAS PROC CORR and coefficients are reported when *p* < 0.1.

## 3. Results

### 3.1. Cocoa Powder (CP) Flavanols

Analysis of the CP fed to pigs showed that the major components were flavanols, theobromine, caffeine, and caffeoyl aspartic acid. The total flavanol concentration was 137.19 mg/g of CP with total flavanols expressed as B-type proanthocyanidin dimer equivalent containing 15.70 mg/g of flavanols up to five polymers as determined by UHPLC HRMS (Table 1).

### 3.2. Clinical Signs

**Figure 1:**
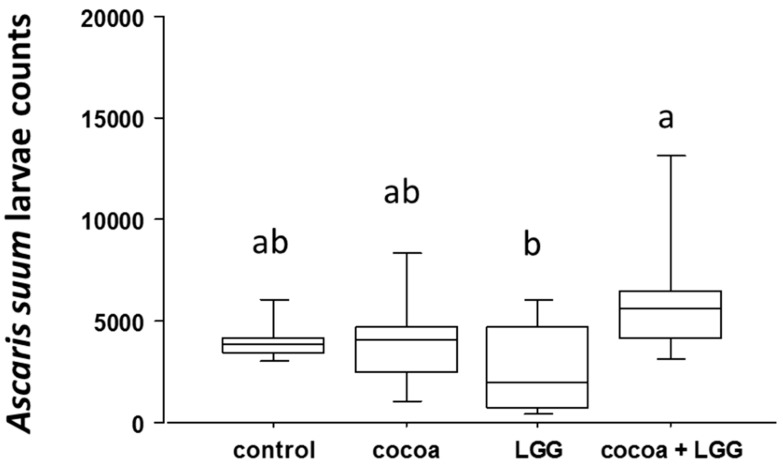
*Ascaris suum* fourth-stage larvae (L4) from intestinal luminal contents 17 days post-inoculation. Data represent mean larvae counts ± SEM (*n* = 8 per treatment). Different letters denote differences among treatments after ANOVA (*p* < 0.05).

There were no clinical signs of disease in any of the pigs. Body weight (BW) increased in pigs fed LGG + CP, 30.7 ± 0.6 Kgs BW (mean ± SE) compared to 28.6 ± 0.8 Kgs BW in control pigs, with no other differences among treatment groups after seven weeks of dietary intervention (*p* < 0.05). Serum profiling of liver enzymes alkaline phosphatase (ALP), alanine transaminase (ALT), aspartate transaminase (AST), total bilirubin, glutamyl transferase (GGT) and total protein were within the physiological range for all four treatment groups. Lactate dehydrogenase (LDH) was elevated in pigs supplemented with LGG + CP (661.75 ± 35.23 U/L) at Week 7 relative to their baseline level (508 ± 38.89 U/L) (*p* < 0.05), but still within the normal physiological range (380–634 U/L) (Table S2). The mean average recovery of *A*. *suum* L4 in pigs fed CP was 4163 ± 908 (mean ± SE), LGG + CP was 6388 ± 1393, placebo control was 4072 ± 888, and LGG was 2700 ± 589. Pairwise mean comparisons indicated a significant increase (*p* = 0.01) in *A*. *suum* L4 in pigs treated with LGG + CP compared to pigs fed LGG (Figure 1).

### 3.3. Abundance of Lactobacillus Rhamnosus in Feces 

**Figure 2:**
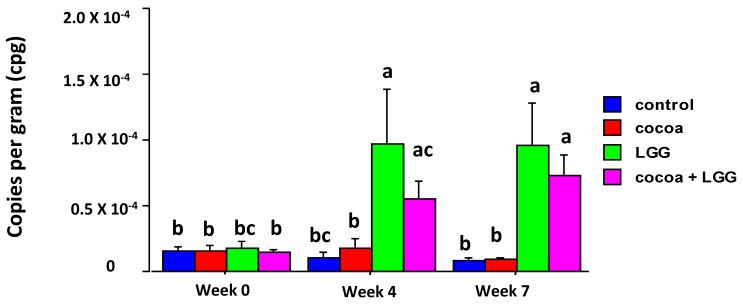
Bacterial LGG abundance in feces of pigs fed different diets. *Lactobacillus rhamnosus* abundance was measured by real time PCR and converted to copy number per gram of feces (cpg). Values shown are means ± SEM (*n* = 8 per treatment). Different letters denote differences among treatments after ANOVA (*p* < 0.05).

The abundance of *Lactobacillus rhamnosus* significantly increased in fecal samples from pigs fed LGG compared to the control pigs at Weeks 4 and 7 (*p* < 0.05), and in pigs fed LGG + CP at Week 7. LGG abundance appeared not to be affected by feeding CP or by infection with *A*. *suum* (Figure 2).

### 3.4. Type 2 Immune Response-Related Gene Expression in the Draining Lymph Nodes

**Figure 3:**
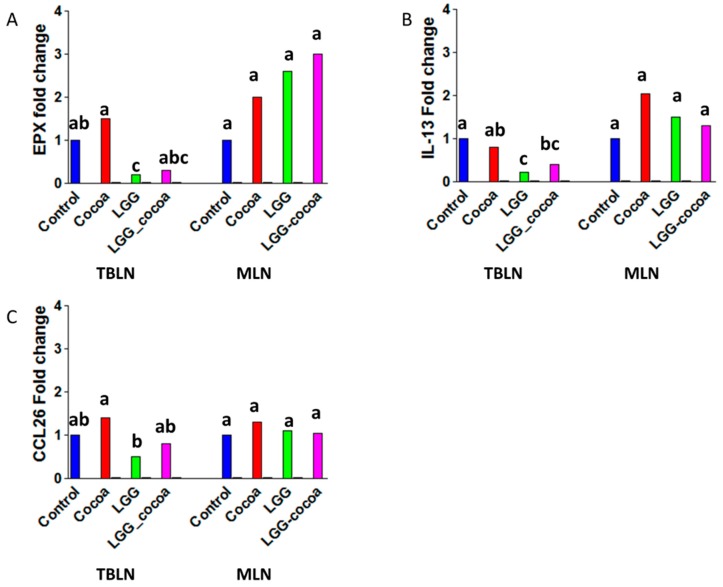
Gene expression related to a type-2 immune response in Tracheobronchial lymph nodes (TBLNs) and Mesenteric Lymph nodes (MLNs). Gene expression in TBLNs and MLNs were measured by RT-PCR (*n* = 8/group): (**A**) EPX (Eosinophil peroxidase); (**B**) IL-13 (interleukin 13); and (**C**) CCL26 (C-C motif ligand 26). Bars represent a mean fold change. The control group was designated as one-fold change. Labeled means without a common superscript letter differ (*p* < 0.05).

Parasitic *A*. *suum* larvae typically show peak migration through the lungs of pigs at seven days after inoculation and begin to appear in the small intestine three days later [23]. Previous studies have shown a prototypical increase in Th2-associated type-2 cytokine gene expression for *EPX*, *CCL26*, and *IL-13* in TBLN [10,19]. In this study, pigs fed LGG and infected with *A*. *suum* had a reduced expression of *EPX*, *CCL26* and *IL-*13 (*p* < 0.05) with no change in gene expression of these three genes when pigs were fed LGG + CP or CP alone. No changes in type-2-cytokine gene expression were observed in MLN draining the small intestine (Figure 3) (data showing ΔCt values and their standard errors along with fold changes are shown in Table S3) or in jejunum tissue (data not shown). 

### 3.5. Toll-Like Receptor Gene Expression in the Draining Lymph Nodes

**Figure 4:**
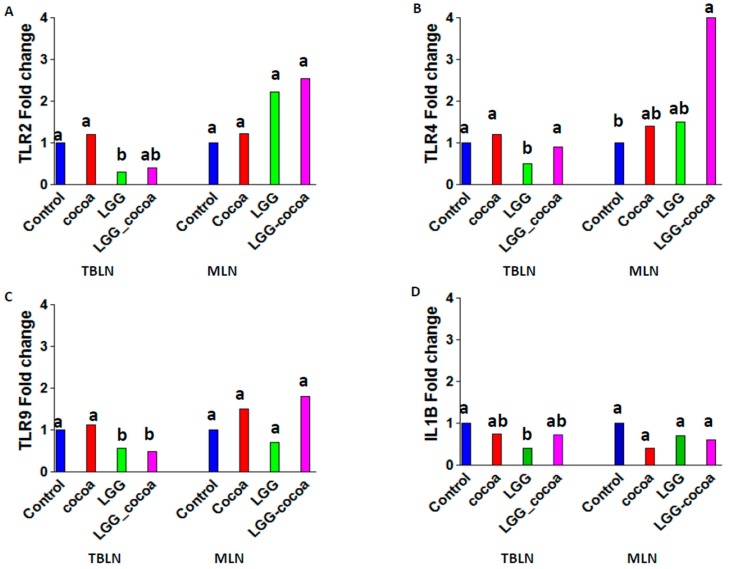
Toll-like receptor gene expression in TBLNs and MLNs. Toll-like receptor (TLR) 2 (**A**); TLR4 (**B**); TLR9 (**C**); and IL-1β (**D**) gene expression in TBLNs and MLNs were measured by RT-PCR (*n* = 8/group). Bars represent a mean fold change. The control group was designated as one-fold change. Labeled means without a common superscript letter differ (*p* < 0.05).

Pigs fed the control diet had levels of TLR2, TLR4, TLR9 and IL1B gene expression in TBLN that were significantly (*p* < 0.05) greater than in pigs fed LGG (Figure 4). In MLN, a significant increase in TLR4 gene expression was observed in pigs fed LGG + CP compared to control pigs (Table S3).

### 3.6. LPS-Induced Alveolar Macrophage (AM) Gene Expression In Vitro

Isolated AM stimulated with LPS in vitro showed a significant increase in TNFα gene expression with no significant difference among treatment groups. No differences in TLR2, TLR4 and TLR9 gene expression were observed (data not shown).

### 3.7. Antibody Response to Ascaris suum

**Figure 5:**
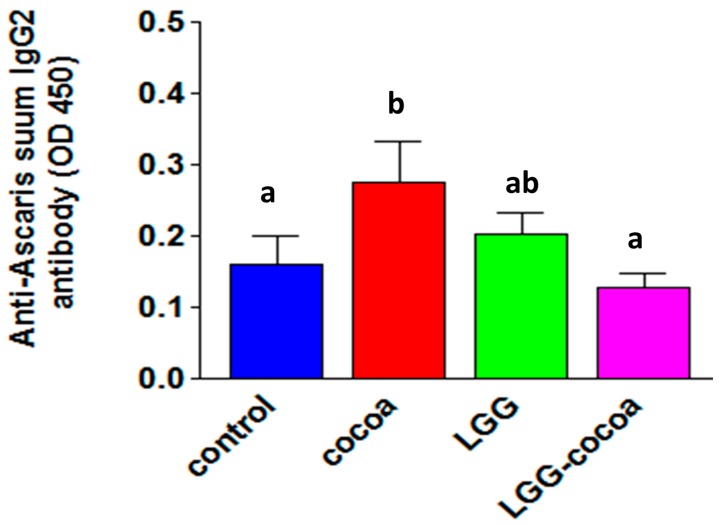
Anti-*Ascaris* IgG2 antibody response. The anti-*Ascaris* IgG2 antibody response was measured using *A*. *suum* proteins extracted from isolated third and fourth stage larvae and serum obtained 10 days post-inoculation with infective *A*. *suum* eggs. Bound serum antibody at a 1/1000 dilution was detected using biotinylated anti-pig IgG2 followed by streptavidin-HRP complex. Values shown are means ± SEM (*n* = 8 per treatment). Labeled means without a common letter differ, *p* < 0.05.

The serum IgG2 antibody response to *A*. *suum* larval antigens was reduced in pigs fed LGG + CP compared to those fed CP alone (*p* = 0.03) (Figure 5). No other differences in anti-*Ascaris* specific IgM, IgG or IgG1 isotype were detected in the serum or ileal content supernatants (data not shown); however, there was a significant inverse correlation between serum IgG1 (r = −0.39, *p* = 0.02) anti-*Ascaris* antibody and *A*. *suum* L4 counts, with a negative but non-significant correlation for serum IgG2 and ileal IgG (r = −0.33, *p* = 0.06) antibody responses and *A*. *suum* L4 counts.

## 4. Discussion

This study provides evidence for an immune modulating effect of daily feeding of *Lactobacillus rhamnossus* LGG on the localized type-2 immediate-type hypersensitivity response in the lungs of pigs infected with *A*. *suum*. *Ascaris suum* is a common and ubiquitous parasitic roundworm that induces a strong type-2-polarized immune response in pigs with significant increases in IL-4, IL-5, IL-13 and other genes in the small intestine, liver, lung and draining MLN and TBLN [10]. The type-2 response is associated with protection against parasitic larvae migrating through the tissues and expulsion from the small intestine between 17 and 21 days after inoculation [10]. Our results showed that pigs fed LGG and infected with *A*. *suum* had reduced levels of IL-13, a key cytokine regulating allergic responses to parasitic infection, eotaxin 3 (CCL26), one of the three related chemokines that specifically activates CCR3 receptor in eosinophils, and eosinophil peroxidase (EPX), suggesting an inhibition of eosinophil recruitment and function in TBLN draining the lungs of *A*. *suum* infected pigs. *Lactobacillus rhamnossus* modulation of type-2 allergic responses has also been reported in a mouse ovalbumin (OVA)-induced allergy model where it suppressed inflammatory cell infiltration in the airways and BAL fluid after challenge with OVA, and reduced IL4, IL5 and IL-13 gene expression in the lungs and spleen [24,25]. In our model, dietary treatment affected type 2 gene expression in the TBLN, but not the MLN draining the small intestine of *A*. *suum*-infected pigs. This may be due to the accumulating numbers and growth of L4 in the small intestine provoking a stronger immune response not sensitive to the bioactive components of the diets tested. In future studies, evaluating diet effects on advanced larval stages or adult *A*. *suum* after longer periods of feeding may prove more relevant to the biology and epidemiology of this infection in pigs and, by extension, humans. 

The role of antibody in protective immunity against *A*. *suum* in pigs is unresolved, as there is a weak association between antibody levels and development of worms several weeks after initial infection [26], and acquired resistance is associated with induction of IgG1 and IgM rather than IgG2 antibodies [27]. In our study, the number of *A*. *suum* L4 were inversely correlated with the serum IgG1 (r = −0.39, *p* = 0.02), serum IgG2 (r = −0.33, *p* = 0.06), and ileal fluid IgG (r = −0.33, *p* = 0.06) antibody response to *A*. *suum* larvae products suggesting some type of humoral immune protection [24]. A comparison of *A*. *suum* L4 detected in the small intestine indicated that pigs fed LGG + CP had more L4 than pigs fed the other diets with a significant difference when compared to LGG fed pigs. Pigs fed LGG + CP also had a significantly lower level of circulating IgG2 antibodies to *Ascaris*-derived antigens when compared to pigs fed CP (*p* < 0.05), suggesting that a weaker antibody response in pigs fed LGG + CP enhanced L4 survival. A negative relationship between levels of anti-*Ascaris* IgG1 antibodies and migrating larvae in the lungs of infected pigs correlated with acquired resistance [27]. Future studies on diet-induced protective responses against *A*. *suum* would target adult worm infection and memory to re-infection.

Our results do not indicate any independent dietary effect of CP or LGG on anti-*Ascaris* antibody production as none of the immunoglobulin isotypes evaluated were changed by diet. Diets with cocoa polyphenols, however, have shown a dose-dependent reduction in OVA specific antibody levels in OVA-sensitized rats [28] or in anti-allergen IgE synthesis in rats fed a conventional cocoa product with high fiber and methylxanthines [29]. Reduced levels of specific serum antibody to *Ascaris* antigens was observed only in pigs fed LGG + CP, suggesting that the synergism of LGG probiotic and bioactive cocoa-derived products may also alter allergic type antibodies [30]. This speculation would be supported when appropriate reagents to detect pig IgE have been developed. The composition of cocoa-flavanols and dose of active ingredients may explain differences in immune modulating effects in different animal models [28,29]. The dose fed in our study was selected to be comparable to that fed to humans [31] where cocoa derived metabolites were reportedly absorbed and distributed to tissues [4]. In addition, factors such as the allergen route for sensitization (i.e., intraperitoneal vs. oral) also have been shown to induce differential responses [8]. 

Feeding LGG to pigs for four weeks increased the abundance of *Lactobacillus* species independent of feeding CP and this increase was not affected by parasitic infection. It also modulated localized type-2 gene expression in response to infection with *A*. *suum* and the levels of pro-inflammatory genes TLR2, -4, and -9 and IL1B without any additional effect of feeding CP. Although feeding CP does not appear to affect the *A*. *suum*-induced type-2 cytokine response, it does synergize with LGG to improve body weight after a seven-week dietary intervention and reduce the anti-*A*. *suum* specific IgG2 response, which may delay the intestinal expulsion of parasitic larvae. Taken together, our data showed that feeding LGG alone reduce expression of genes associated with the type-2 immune response and inflammation in the lymph nodes draining the lungs with a lower yield of intestinal parasitic L4 compared to pigs fed LGG +CP. However, feeding LGG + CP can reduce IgG2 antibody levels, expression of some genes linked to parasite-induced inflammation and improve growth. The impact of these features would require a longer interaction with the infection to confirm the health benefits of feeding these diets.

## Figures and Tables

**Table 1:** Chemical composition of Acticoa cocoa powder.

Measure	Concentration, mg/g
Total Flavanols ^a^	137.19
Flavanols (up to 5P) ^b^	15.7
Individual flavanol	
Catechin	2.87
Epicatechin	4.27
Procyanidin B2	3.7
Other B-type dimer	0.95
Procyanidin C1	2.72
Other B-type trimer	0.43
Other B-type tetramer	0.56
Other B-type pentamer	0.2
Theobromine	19.26
Caffeine	1.45
Caffeoyl asparitic acid	1.83

^a^ PAC result from 4-dimethylaminocinnamaldehyde(DMAC) colorimetric method is expressed as B-type proanthocyanidin, dimer equivalent of milligram PACs content per gram of cocoa powder. ^b^ Cocoa powder methanol extract was injected into UHPLC-Orbitrap MS system. Catechin/Epicatechin, procyaidin B1 and procyanidin C1 used as standards for monomers, dimmers and trimers, respectively. Tetramers and pentamers were measured according to previously published procedures using (-) cathechin (Sigma Aldrich, St. Louis, MO, USA) as reference standard with relative response factors.

## Supplementary Materials

The following are available online at www.mdpi.com/2072-6643/9/10/1113/s1, Table S1: Pig Diet; Table S2; Biochemical serum markers at Week 7; Table S3-ΔCt values and their standard errors along with fold changes.
