# Supplementary material for: Flavanol-Rich Cocoa Powder Interacts with Lactobacillus rhamnossus LGG to Alter the Antibody Response to Infection with the Parasitic Nematode Ascaris suum
Source: Nutrients. 2017 Oct 12;9(10):1113. doi: 10.3390/nu9101113 (PMC5691729; doi:10.3390/nu9101113)
Supplement: Supplementary file 1 [file nutrients-09-01113-s001.zip › nutrients-217728-supplementary.pdf]

Online Support Materials

Table S1. Pig diet

Nutrients	g/ kg diet	
Protein	153.2	
Fat	26.1	
Fiber	34.1	
Total Kcal/kg diet	2957 kcal	
**Ingredients**	**g**	
Corn	718.9	
48% Soybean meal	198.8	
Alfalfa meal 17%	50.0	
Salt	5.0	
Calcium Carbonate	5.8	
[1]Swine trace mineral	1.0	
[2]Swine vitamins	1.0	
Selenium premix	0.5	
Choline 60%	0.3	
Soybean oil	2.5	
Monocalcium phosphate	16.3	
Total	1000	
**Dietary Treatment**	**Treatment**	
Cocoa powder (g/d)	0	26
[3]Dextrin (g/d)	1.24	0
[4]Cellulose (g/d)	6.42	0

[1] Swine trace mineral (PIGMASTER G.F 50 from ADM Alliance Nutrition) per kg: Calcium 22%- 26.4%, Phosphorus 6%, Salt 12% -14.4%m, Selenium 12 ppm, Zinc 2450 ppm

[2] Swine vitamins (SWINE VITAMIN PREMIX from DSM) per kg: Vitamin A 4,409,171 IU, Vitamin D3 881,834 IU, Vitamin B12 44 mg, Riboflavin 8,818 mg, d-Pantothenic Acid 17,637 mg, Niacin 30,864 mg

[3, 4] Dextrin and cellulose were added to match total soluble and insoluble fiber of cocoa powder.

Table S2. Biochemical Serum markers at week 7

Index	Treatment	wk 0	wk 7	p-value
ALP	Control	158.14±24.19	151.71±22.35	
118-395 U/L*	LGG	184.77±15.19	195.66±18.22	
	Cocoa	150.62±12.09	136.50±8.89	
	LGG+Cocoa	180.25±18.39	162.62±14.19	
ALT	Control	54.86±5.47	60.28±3.60	
31-58 U/L	LGG	47.33±3.31	50.33±6.41	
	Cocoa	47.75±3.89	46.87±5.55	
	LGG+Cocoa	44.12±2.66	54.62±5.30	
AST	Control	33.86±4.50	27.57±2.39	
32-84 U/L	LGG	38.22±4.31	34.89±4.37	
	Cocoa	35.62±4.74	28.62±3.36	
	LGG+Cocoa	30.63±4.52	30.13±2.65	
Total bilirubin	Control	0.129±0.02	0.100±0.00	
0-10 mg/dL	LGG	0.122±.0.01	0.156±0.02	
	Cocoa	0.125±0.02	0.138±0.02	
	LGG+Cocoa	0.100±0.00	0.163±0.02	0.004
LDH	Control	482±28.35	610±71.4	0.021
380-634 U/L	LGG	593±33.60	684.78±69.41	
	Cocoa	525.38±22.74	622.75±51.31	
	LGG+Cocoa	508±38.89	661.75±35.23	0.011
GGT	Control	38±1.77	43.28±3.48	
10-60 U/L	LGG	41.22±3.14	42.22±3.64	
	Cocoa	38.87±2.92	39.87±3.23	
	LGG+Cocoa	39.63±2.32	44.50±3.92	

Total protein	Control	6.60±0.21	8.50±0.31	0.003
7.9-8.9 g/dL	LGG	7.0±1.96	7.87±0.46	
	Cocoa	6.65±0.24	7.20±0.53	
	LGG+Cocoa	6.65±0.15	7.72±0.26	0.003

* Indicate normal values expressed in U/L for ALP,ALT,AST,LDH,GGT enzymes and mg/dL for bilirubin AST= Aspartate Transaminase; LDH=Lactate Dehydrogenase;
or g/dL for total proteinaline Phosphatase; ALT= Alanine Transaminase; AST= Aspartate Transaminase; LDH=Lactate Dehydrogenase; ALP=Alanine Phosphatase
ALT= Alanine Transaminase; AST= Aspartate Transaminase; LDH=Lactate Dehydrogenase; GGT=Gamma-glutamyl transferase

Table S3. Dietary induced gene expression changes in lymph nodes

		Dietary Intervention										
		Control		Cocoa		LGG		LGG + Cocoa				
		mRNA expresion ΔCt (mean ± SD)								Main effects [3]		
GENE		ΔC_T [1]	Fold of control	ΔC_T Cocoa	Fold relative to control [2]	ΔC_T	Fold relative to control [2]	ΔC_T	Fold relative to control [2]	Diet	Treatment	Diet* Treatment
EPX	TBLN	12.82 ± 0.61 ^{ab}	1	12.30 ± 0.61 ^{a}	1.5	14.85 ± 0.61 ^{c}	0.2	14.31 ± 0.61 ^{abc}	0.3	0.402	**0.003**	0.991
	MLN	16.53 ± 0.73	1	15.53 ± 0.73	2	15.20 ± 0.73	2.6	14.94 ± 0.73	3	0.397	0.201	0.613
CCL26	TBLN	3.87 ± 0.33 ^{ab}	1	3.38 ± 0.33 ^{a}	1.4	4.73 ± 0.33 ^{b}	0.5	4.21 ± 0.33 ^{ab}	0.8	0.133	**0.016**	0.957
	MLN	4.47 ± 0.48	1	4.11 ± 0.48	1.3	4.31 ± 0.48	1.1	4.40 ± 0.48	1	0.783	0.892	0.644
			1									
IL-13	TBLN	13.38 ± 0.41 ^{a}	1	13.59 ± 0.41 ^{ab}	0.8	15.48 ± 0.41 ^{c}	0.2	14.69 ± 0.41 ^{bc}	0.4	0.493	**0.001**	0.231
	MLN	14.08 ± 0.69	1	13.05 ± 0.69	2	13.48 ± 0.69	1.5	13.68 ± 0.69	1.3	0.555	0.982	0.380
TLR2	TBLN	8.85 ± 0.53 ^{a}	1	8.58 ± 0.53 ^{a}	1.2	10.50 ± 0.53 ^{b}	0.3	10.04 ± 0.53 ^{ab}	0.4	0.505	**0.008**	0.860
	MLN	11.90 ± 0.68	1	11.59 ± 0.68	1.2	10.75 ± 0.68	2.2	10.56 ± 0.68	2.5	0.719	0.120	0.935
TLR4	TBLN	7.82 ± 0.40 ^{a}	1	7.59 ± 0.40 ^{a}	1.2	9.03 ± 0.40 ^{b}	0.5	7.88 ± 0.40 ^{a}	0.9	0.097	0.072	0.259
	MLN	9.30 ± 0.85	1	8.79 ± 0.85	1.4	8.67 ± 0.85	1.5	6.80 ± 0.85	5.6	0.172	0.136	0.427
											mean ±SD)	
TLR9	TBL	9.83 ±	1	9.65 ±	1.1	10.92 ±	0.5	10.85 ±	0.5	0.736	**0.004**	0.872

	N	0.36^{a}		0.36^{a}		0.36^{b}		0.36^{b}				
	MLN	14.26 ± 0.81	1	13.67 ± 0.81	1.5	14.70 ± 0.81	0.7	13.42 ± 0.81	1.8	0.264	0.908	0.675
IL1b	TBLN	12.75 ± 0.43^{a}	1	13.18 ± 0.43^{ab}	0.7	14.13 ± 0.43^{b}	0.4	13.19 ± 0.43^{ab}	0.7	0.5643	0.1191	0.1251
	MLN	13.28 ± 1.07	1	14.36 ± 1.07	0.4	13.80 ± 1.07	0.7	14.06 ± 1.07	0.6	0.5358	0.9226	0.7084

[1] Data represent ΔCt values after gene expression was normalized to the housekeeping gene (Ct gene-Ct houseeepng gene) for each dietary treatment

[2] Changes in gene expression expressed as fold change compared to the control group which was designated as 1 fold change.

[3] Data represent p-values associated with the diet, treatmment or Diet * Treatment effects from the normally distributed ANOVA model
